# A Survey of Datasets, Preprocessing, Modeling Mechanisms, and Simulation Tools Based on AI for Material Analysis and Discovery

**DOI:** 10.3390/ma15041428

**Published:** 2022-02-15

**Authors:** Imran, Faiza Qayyum, Do-Hyeun Kim, Seon-Jong Bong, Su-Young Chi, Yo-Han Choi

**Affiliations:** 1 Department of Computer Engineering, Jeju National University, Jeju 63243, Korea; imranjofficial@jejunu.ac.kr (I.); faizaqayyum@jejunu.ac.kr (F.Q.); 2 Research Center of Advance Technology, Jeju National University, Jeju 63243, Korea; 3 Department of Artificial Intelligence, Korea University of Science and Technology, 217, Gajeong-ro, Youseong-gu, Daejeon 305-370, Korea; sunjong@ust.ac.kr; 4 Electronics and Telecommunications Research Institute, Dajeon 305-370, Korea; chisy@etri.re.kr (S.-Y.C.); tabby@etri.re.kr (Y.-H.C.)

**Keywords:** material datasets, material data pre-processing, modeling mechanisms, AI simulations tools, material analysis

## Abstract

Research has become increasingly more interdisciplinary over the past few years. Artificial intelligence and its sub-fields have proven valuable for interdisciplinary research applications, especially physical sciences. Recently, machine learning-based mechanisms have been adapted for material science applications, meeting traditional experiments’ challenges in a time and cost-efficient manner. The scientific community focuses on harnessing varying mechanisms to process big data sets extracted from material databases to derive hidden knowledge that can successfully be employed in technical frameworks of material screening, selection, and recommendation. However, a plethora of underlying aspects of the existing material discovery methods needs to be critically assessed to have a precise and collective analysis that can serve as a baseline for various forthcoming material discovery problems. This study presents a comprehensive survey of state-of-the-art benchmark data sets, detailed pre-processing and analysis, appropriate learning model mechanisms, and simulation techniques for material discovery. We believe that such an in-depth analysis of the mentioned aspects provides promising directions to the young interdisciplinary researchers from computing and material science fields. This study will help devise useful modeling in the materials discovery to positively contribute to the material industry, reducing the manual effort involved in the traditional material discovery. Moreover, we also present a detailed analysis of experimental and computation-based artificial intelligence mechanisms suggested by the existing literature.

## 1. Introduction

The need for artificial intelligence (AI) applications in the simulation and exploration of novel ceramic materials is increasing. Materials design based on AI analysis is foreseen to lead to innovative materials and reduce the development cost in terms of time and resources. However, the scientific community identified many limitations of advanced materials discovery and application based on AI and advanced machine learning techniques. For instance, there are several issues related to computational simulation, and the structures of the materials involved require high-performance index properties. Therefore, advanced materials research based on the convergence between AI techniques and experimental mechanisms is needed to produce the basic comprehension of the input parameters conditions and performance index properties. In the first step, raw data is prepared for model building using pre-processing and feature engineering techniques. The second step is building an AI model using AI-based learning algorithms. Finally, the performance evaluation of the model and interpretation of the model knowledge into input parameters and performance index properties for assessment of the materials.

The combination of machine learning and AI has brought an immense revolution in various walks of life, including varying materials detection. Initially, symbolic methods were preferred to extract hidden knowledge from the data. Later on, the techniques were tuned to incorporate some advanced functionalities in artificial neural networks that own self-learning capabilities with the help of neurons [1]. Similarly, several other useful machine learning models, including decision trees (DTs) [2], and support vector machines (SVMs), etc., were also introduced. Presently, various novel machine learning-based models like deep learning to analyze a massive amount of data have grabbed the attention of industry and academia [3,4,5]. Thus, the process of analytical building formulation becomes automated with the help of the machine learning paradigm. The effort of explicit programming to reveal the hidden patterns from the data has been diminished with the initiation of machine learning algorithms that learn from the data iteratively. Different machine learning methods effectively handle high-dimensional data, including clustering, regression, and classification. The prime focus of machine learning-based models is the extensive scrutinization of massive databases to extract hidden knowledge. Machine learning (ML) models learn from the historical data to return accurate and reliable results in diversified fields, including image recognition, natural language processing (NLP), and information security. Many routine activities like web searches, fraud detection, next-best offers, sentiment analysis are addressed by machine learning-based models [6]. 

To date, the scholarly community in materials science has made tremendous efforts in terms of collecting an immense amount of data pertaining to materials property to stipulate access to relevant personnel with the open quantum materials database (superconducting), inorganic crystal structure database (ICSD)), superconducting critical temperatures (SuperCon), etc. [7]. Furthermore, a pioneering study pertaining to machine learning in material science was coined in the 1990s amid the era of harnessing artificial neural networks and symbol methods to predict the ceramic-matrix composites for tensile and compressive power of the fiber interfaces the pattern of corrosion [8]. Likewise, the machine learning paradigm has played an immense role in addressing diverse aspects related to material science, including material property prediction and new materials discovery. 

Let us consider a definition of machine learning in terms of material science as <C, W, E>, C, W, and E denote conduct, work, and experience. According to the vital concept, a computer program learns from the experience E with the corresponding work/task W, as measured by a Conduct measure C if its performance on Works in T, as by C, enhances along with the experience. Typically, a machine learning model may be constructed while harnessing machine learning to handle an issue about materials science. Following is the general demonstration of such a machine learning model. The prevailing paradigm of such machine learning systems is given as follows:

In this example, the final goal indicates the said issue, which is typically denoted with the help of an objective function. Similarly, according to some pre-defined method, the sample represents the subset of the population picked for analysis [9]. Typically, data processing parts such as data cleaning and feature engineering form actual data into the sample data. The first step, i.e., data cleaning, locates noisy patterns in the data and prepares it for experimentation by removing all the abnormalities from the data [10]. The later part, i.e., feature engineering that includes feature construction, feature extraction, feature selection, and feature learning, is comprised of harnessing domain knowledge pertaining to data in order to form features that machine learning models can process. The feature engineering process is quite complex in nature. The models comprise of varying machine-learning algorithms and different optimization algorithms. The widely employed machine learning models include SVM, artificial neural networks (ANN), and DTs and the optimization algorithms include particle swarm optimization (PSO), genetic algorithms (GAs), and simulated annealing algorithms (SAAs), to name a few. The model denotes characteristics of a system in the form of mathematical formulations and points to the algorithm applied to the sample data. 

Material applications based on AI mechanisms have emerged recently with increased experimental and computational data [11]. One important task of material science-based AI applications is to use existing material data to predict the properties of new materials by using data science methods and mathematics [12]. The first step is to build a descriptor model that can predict the property of interest based on a known set of input material-specific features. For instance, one of the vital descriptor models where input variables are material structure features is called quantitative structure-property relationship (QSPR). In the input and output of material properties, a complex relationship is challenging to handle by traditional linear and non-linear correlation methods. However, these complex relationships can now be efficiently modeled by ML methods [13,14]. After performing descriptor modeling analysis and familiarizing with data, a model is applied to predict material behavior using material analysis models based on AI or statistics. ML models are intelligent and improve performance automatically by experience using training data to find patterns from data. In summary, using AI methods in materials science has received a significant concentration from researchers. AI and its subfields, such as machine learning, have proven as excellent techniques for analyzing big data retrieved from material databases and datasets. The upcoming sections of this review paper summarize the datasets available for material science applications, data pre-processing and AI-based modeling techniques, and materials simulation tools. AI mechanisms such as deep learning show significant improvements and potential for predicting compositions, processes conditions, and performance properties of materials to reveal the changes in specific parameters for modeling materials behavior. Moreover, this study discusses AI techniques, showing the significance of advanced AI mechanism-based simulation models in designing and optimizing properties prediction for advanced materials discovery. 

The rest of the paper is organized as follows: a brief discussion on datasets and data pre-processing for material modeling is presented in Section 2. Section 3 presents the methods and mechanisms used for material discovery and analysis. Simulation results and tools are discussed in Section 4. Section 5 presents the commonly used machine learning application in the material science industry. Finally, the conclusion and future work directions are presented in Section 6.

## 2. Datasets and Data Preprocessing for Material Modeling

AI is an exciting technique to predict Material discovery, and It has been used to predict material properties in an eco-friendly and effective manner. However, there is a lack of benchmark datasets in this field, especially those encompassing parameters for material discovery such as task, size, and material systems deemed import indicators for material information discovery. Therefore, selecting optimal AI models, model architecture, data featurization, data splitting, including algorithms for a given task is challenging. The contemporary state-of-the-art in material sciences formed a data repository of fifty different data sets and revealed a minimal quantity of such data sets. The material system-based framework comprises varying material researches, and the data set contains computational and experimental data, classification, and regression data.

These benchmark data sets are used as a baseline to form other comprehensive benchmark data sets in the future for comparing AI-based models. Various material properties and experimental and calculated values are included because the data is from the past literature. The data collection compares AI models for material informatics efficiently and accurately and improves computational materials science practices. The combination of all the benchmark data sets into an individual repository encompassing material data for probabilistic and AI methods is described in the publications. It also enables researchers to compare AI-based models, such as ML, which helps find an efficient material discovery method. In the generalization of ML models, a diverse dataset allows researchers to investigate quickly. Therefore, to test several ML approaches and enhance the diversity and types, some data from the MatBench project are included and can be used as a repository that can be readily obtained for ML and AI-based learning techniques. The source from which these datasets are collected is based on existing literature [13,15].

Table 1 presents Summary of datasets based on names, material properties, dataset size, and AI tasks. Methods used for ML and AI in materials science literature were used to collect the categorical dataset population of the dataset. Above each color bar, descriptors are listed, to the left categorization methods used in the graph, and each bar describes the number of datasets. The size of the data set varies between 100 to 5000 instances encompassing the small and large data set having 100 and 1000 instances, respectively. The Data Type category has calculated data and experimental data belong to computed data, which means a large amount of data used from this data belongs to computed data [16,17,18,19,20,21,22]. 

The listed data sets are explained according to four characteristics: Dataset name represents the name of the data, Material Property explains the associated properties such as Curie Temperature (Tc), lonic Dielec Const., etc. In addition, each dataset is cited with its source research paper and its name. In addition, each dataset is cited with its source research paper and its name [23,24,25,26,27,28]. 

### Data Preprocessing for Material Modeling

For analysis, it is required to structure data from raw data collected. The data set is in three different formats, i.e., structured, semi-structured, and unstructured. Highly organized and neatly formatted data belong to structured data since they are in tabular format feasible to retrieve, process, and maintain. The structured data set is formed using mediums such as excel sheets or sensor data. In contrast, unstructured data is not organized in a pre-defined format; raw form, irregularities, and disorganization result in complex processing forms. The samples for unstructured data encompass the data formed using IoT sensors, video, audio, and images. At the same time, transpose, join and pivot functions can be employed to convert raw data into a structured format.

The exploratory data analysis and pre-processing phase of material discovery modeling play a valuable role in revealing the exciting insights from the data. The prime focus of data analysis is to identify the data trends employing different statistical techniques, recapitulating data numerically and graphically. For example, the key features of central tendency, spread such as standard deviation and variance, can be obtained with the above analysis. Moreover, key features, including outlier detection and distribution shape, may be ascertained.

Two types of outliers that can be removed from the dataset, i.e., technical errors or data entry errors and incorrect data values, can influence and skew the data. Box plots, scatter plots, or other graphs can graphically visualize statistical outliers. Visualization software such as Matlab, R Programming, Python, Microsoft Power BI, and Microsoft Excel can perform EDA. This statistical procedure, such as graphical visualization techniques, assists in summarizing data using visualization charts such as histogram, multi-variate chart, scatter plot, Boxplot, and histogram.

Every year, expeditious data growth in material science is witnessed, which makes the quantity of data double; this is also considered one of the primary reasons for evolving the paradigm of material science in diverse disciplines [29]. However, the growing data rate in material science is challenging. For instance, data analysis, processing, collection, indexing, storage, and retrieval are quite complex procedures for handling extensive material science data. Therefore, it is essential to address the issues mentioned above in material science to extract hidden patterns and new material discovery for visualization, predictive approach, analysis, and better data storage [30].

**Figure 1:**
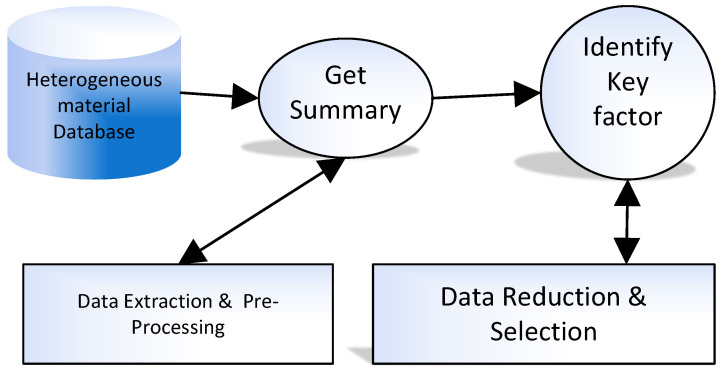
Key steps in material data Pre-processing.

Furthermore, appropriate data storage plays an immense role in the analysis of characteristics of materials’ characteristics [31]. In material science, the most amount of the data is formed using new calculations or experiments; therefore, there is a dire need for data-driven based models to perform material discovery and deployment. Thus, analyzing properties and trends of data and discovering new materials data analysis has a primary role in material science. Data in material science get collected and compared with existing data while the researchers generate a large amount of data through experiments and simulation. Figure 1 presents key steps in material data pre-processing for data preparation.

For better analysis, it is essential to understand the format and representation of data because it is stored in several structures in the database to perform certain pre-processing and ensure data quality [32]. The pre-processing techniques include conversion of attribute type, sampling, feature extraction, feature selection, and data discretization used to remove noisy values, missing data, outlier data, duplicated data, etc., and remove unsuitable data. Furthermore, the technique can be either supervised or unsupervised [33].

Supervised learning for predictive modeling can be used to pre-process data that can be used for modeling. However, splitting the datasets needs extra care to avoid overfitting that shows the model’s accuracy. Depending on the target attribute, a regression can be applied if it is numeric, classification technique can be used if it is categorical [34]. The ensemble learning technique is another approach that combines various methods to maintain accuracy. The data mining approach can also be used instead of predictive modeling for relationship mining and clustering to find hidden patterns associated with data. Data pre-processing is deemed a challenging process in Big Data analysis and management because of the extensive data, variety, and velocity gathered from heterogeneous sources. Tool selection and rule discovery are two tools explored to address these issues. Many mechanisms have been proposed to pre-process data and their characteristics; however, it is difficult to pick the best-suited data sets depending on the data type. Likewise, the rule-based discovery also handles data processing to some extent; however, the issue arises in deciding the number of rules for specific data collection.

In general, crystal structure and bond strength are two factors on which material property depends; for this reason, feature identification strongly correlates with a material property of interest is complex to be applied for machine learning procedures. Thus, a good material descriptor meets the following three criteria. (1) A unique characterization of the material, (2) Sensitive to the target property, and (3) easy to obtain. In addition, descriptors at different levels of complexity can be defined depending on the problem or property being studied [34].

An example is a molecular design; If the boiling point of a nonpopular organic compound is analyzed, a gross level can be considered as the definition of descriptor, like total molecular weight wherein the focus is on the prediction of dielectric constant, the descriptor should include atomic-level information. A plethora of essential descriptors has been recapitulated in [35]. One-dimension descriptors are the simplest, such as weight and surface area, molecular volume, number of electrons, and non-polarities that carry little or no information about the structures of the materials. As discussed before, a preferable structure for predicting specific properties is descriptors with two or three dimensions. The topological descriptors contemplate the material’s 2D graphic structure and show branching, symmetry, and atom connectivity [36].

The most common topological descriptors are the adjacency matrix and the connectivity index [37]. Still, they do not contain any stereochemistry information, which is a limitation. 3D materials descriptor is important Radial Distribution Function (RDF) expressed as g(r) denotes probability for identifying particle at a distance from some other atom. The empirical measurements like ab initio calculations and X-ray computations obtain the descriptor type. Therefore, it becomes mandatory to investigate the high dimensional data sets along with the reduction in dimensionality tool before constructing ML models. Many algorithms exist to reduce dimensions of the feature space and identify relevant descriptors. The algorithms include linear discriminant analysis (LDA), Principal component analysis (PCA), and multidimensional scaling (LDA). For example, using orthogonal transformation, the combination of correlated values is converted into a minimized set of uncorrelated new variables using PCA, deemed as principal components (PCs). PCS results in minimized dimensional space that depicts the original data. For example, four-dimensional space was formed by reducing 12-dimensional solvent descriptor space by Zhou et al. [38].

The existing techniques of data collection and pre-processing mechanisms for the collected data provides these key directions for future research:

- New publicly available database construction tools can be developed based on existing datasets.
- The datasets should be made available through open-source programming language-based libraries and APIs.
- Advanced pre-processing-based authoring tools and mechanisms should be introduced.
- The problem of small datasets can be addressed through AI-based data augmentation mechanisms.

Advanced data imputation mechanisms can address the problem of missing material data.

## 3. Modeling Mechanisms

The expeditious rise in computational and experimental data has emerged in the field of material informatics (MI) [39]. A necessary MI process utilizes existing data sets to form a predictive model for new materials’ discovery harnessing mathematics and information science procedures [40]. The main focus is on developing a descriptor model which predicts the property of interest using a set of input material-specific features. The vital descriptor is one in which input parameters denote material structure features as QSPR.

In the input and output of material properties, a complex relationship is challenging to handle by traditional linear and non-linear correlation methods. These crucial associations may effectively be modeled using machine learning techniques [41]. After familiarizing and conducting EDA and data, a model is utilized to predict the rest of the useful life, wherein the machine fails.

Big Data with the predictive algorithm can perform better analysis in material science. However, predictive algorithms do not match with most of the theories of material science, but material science may be moved. The model interacts with a dynamic environment to maximize a reward function and does not need to be labeled input/out pairs to be available compared to Supervised ML. An example of this approach is the Markov decision process or Q-learning technique. On the other hand, ML needs to make a model, form training samples, and manage metadata for predictions. ML is deemed a significant component of AI, which derives models prepared using historical data. Also, it has a vital role in material science because it can reveal hidden data patterns regardless of having information about the underlying mechanism. The built machine learning models can be employed for design and material discovery.

**Figure 2:**
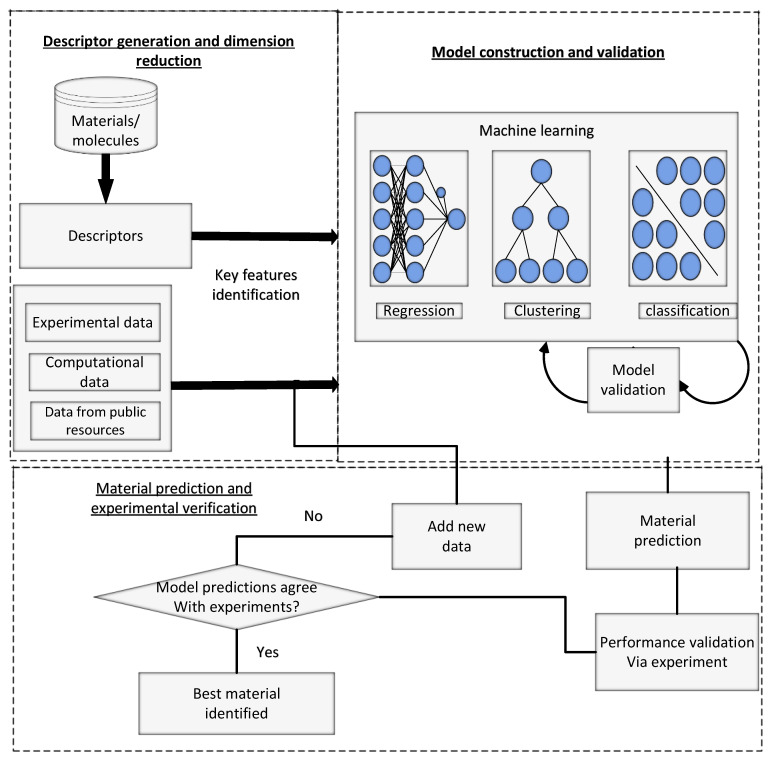
Generic workflow for materials discovery and design based on ML.

The machine learning models designed for material studies include prediction of mechanical and physical properties of alloys, steel fatigue strength, catalytic activities, electronic bandgaps of perovskite materials, and acid dissociation constants, along with finding promising porous materials [42] mixed oxide catalysts [43], and photovoltaic materials [44]. Material design and discovery using machine learning-based workflow is shown in Figure 2. Three primary steps involved are: First, a descriptor is generated, and data dimensionality is reduced. The second step is prediction and verification of new predicted data using experimental verification mechanisms. The third step is model building and validation.

The first phase involves utilizing features or descriptors to show materials in a dataset. This step needs certain background information regarding the application’ and class of materials. In the second phase, the model is mapped among target properties and descriptors using known data against a composition of reference materials.

**Figure 3:**
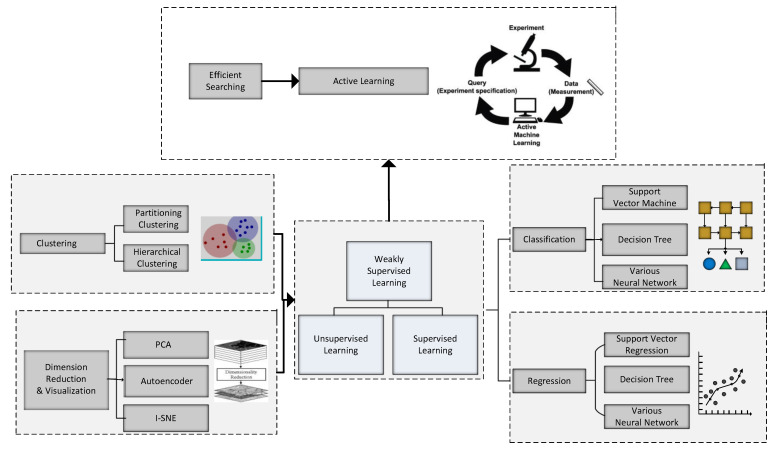
The Structure of ML methods reviewed for supervised learning-based modeling.

Many machine learning techniques comprising linear and non-linear regression can be adopted for mapping. Finally, inverse design is performed based on the ML models in the last step to find new materials with desired properties. The performance of the most promising candidates can then be verified experimentally. As explained earlier, the categories of machine learning models are segregated into unsupervised and supervised learning models. Figure 3 presents the structure of ML methods reviewed for supervised learning-based modeling. The supervised models are further divided into two parts, classification, and regression.

The supervised learning models find a function that has a capacity for novel material discovery using known materials and their properties. On the other hand, if the property to be targeted comprises a continuous value, it falls under the regression category. In this regard, the widely employed regression models include ANNs, Kriging, and SVMs [45]. If the target outputs are discrete, then it is classification. Commonly used classification algorithms are the decision tree [46] and random forest [47].

Supervised learning finds a function that predicts the target class by mapping the input variable to the output property, whereas association among data instances is discovered in unsupervised learning. The clustering technique divides a data set into different groups so that similar data instances or those having a little distance are grouped into the same cluster. The clustering models can be pretty valuable in revealing physical information from the data and identifying novel material discovery using contemporary models [48]. Hierarchical clustering, K-means [49], and hidden Markov modeling [50] are popular clustering algorithms.

**Figure 4:**
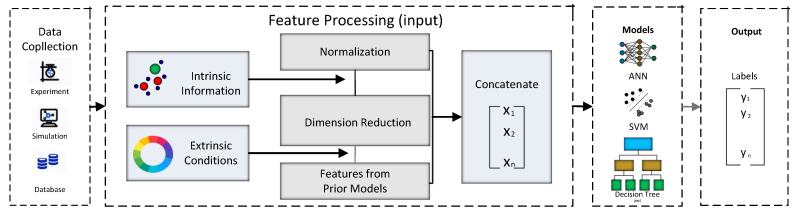
The framework of supervised learning with two main categories of features.

The applications are utilized during the complete life cycle of the Material discovery process [51]. The study has critically assessed the role of machine learning tools for material discovery and relevant advanced concepts utilized by many ML techniques. However, a minimal number of studies have delineated AI or ML-based review studies in the context of material discovery. Moreover, most of them have primarily focused on individual techniques or single-material systems. Therefore, AI-oriented material discovery has received the scientific community’s attention in terms of application-based context. The analysis of the contemporary AI-enabled material detection has broadly been categorized into characterization, property prediction, and theory paradigm discovery. In addition, the models holding potential for material discovery and future challenges have also been addressed. A valuable combination of different AI-enabled models is the prime focus of this study. Figure 4 presents the framework of supervised learning with two main categories of features.

The dataset can be an existing database or generated from laboratory experiments or simulations. Features in material discovery are two intrinsic and extrinsic information; the pioneer step of the pre-processing includes normalization; the second one is to reduce the dimensionality of the data to filter less valuable features. In the end, a few of the raw features from the previous models are tuned and assigned to the final predictive model.

**Figure 5:**
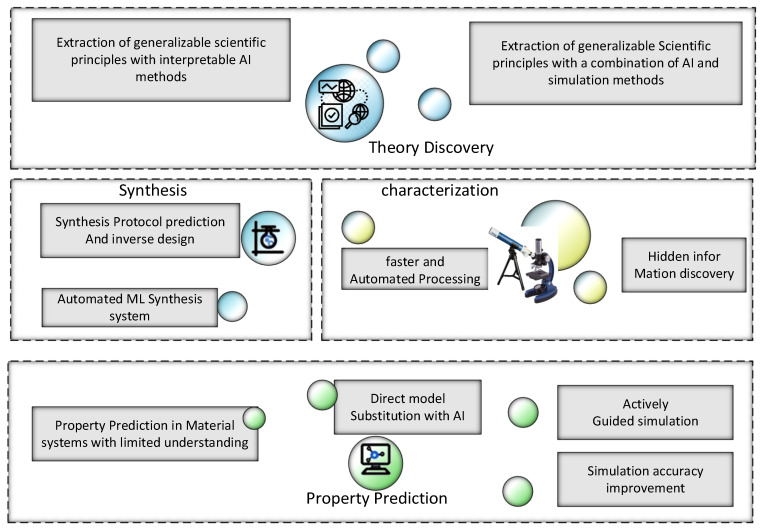
AI applications through the whole life cycle of material discovery.

To ensure which feature is suitable for a model to perform well is the most critical step. A system encompassing two primary classes of features related to supervised learning is delineated below; the system represents supervised learning having two kinds of parameters [52]. The material discovery process has four major parts, i.e., characterization, property prediction, synthesis, and theory of paradigm discovery. The structure of AI applications with the life cycle of material discovery is given in Figure 5. ML for material science envisions automated identification of key data relationships and gaining scientific understanding. 

**Figure 6:**
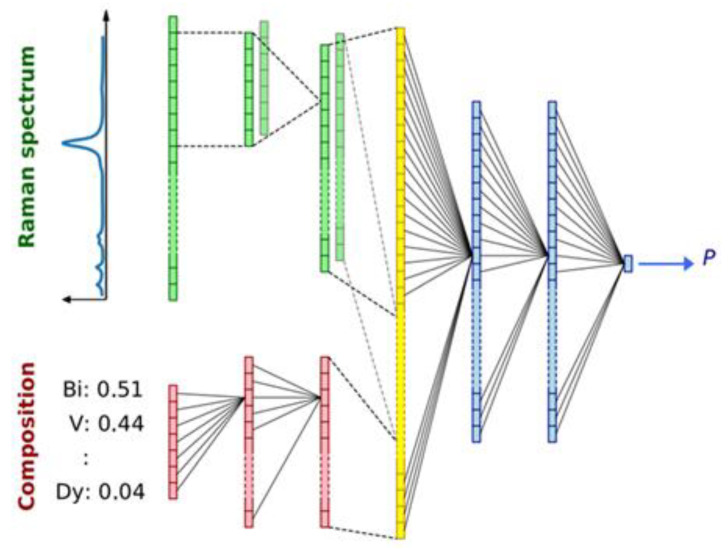
Schematic of CNN model structure takes the Raman spectrum and the composition as input to predict P.

A neural network is harnessed to reveal hidden patterns from the data; in other words, it can be deemed as a process of devising relevant knowledge from the data rather than considering an application as a prediction tool. CNN-based neural networks models are widely utilized wherein complex data is modeled into multidimensional as done by combinatorial material science experiments. Forming a model to analyze the prediction output out of materials, the following assessment of the trained model in the context of gradients signifies primary data relationships, and interpreting these relationships by humans results in fundamental understanding based on a model trained using ML-based relevant strategies. Figure 6 presents a schematic of the CNN model structure that takes the Raman spectrum and the composition as input for prediction. The layers depicted using varying colors indicate different purposes. For instance, red colors show the dense layers acting on composition, and green color represents the convolutional 1D layer processing spectral and composition data.

**Figure 7:**
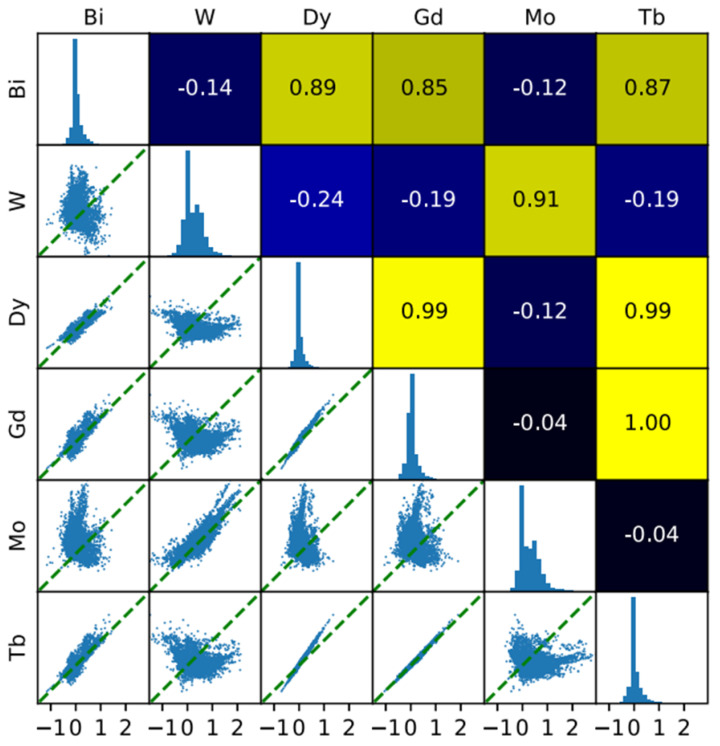
Pairwise correlation analysis of gradients for six composition dimensions of the input data.

Figure 7 presents pairwise correlation analysis of gradients for six composition dimensions of the input data. A systematic CNN-based material discovery model considers composition and Raman spectrum-based parameters for predicting P. Since the V has inherited the disadvantage of having an inverse correlation with all the other data points in the left-most area below and Bi, V was filtered out. A correlation plot represents gradient pairs for an individual sample over the eight frameworks. Each plot on the diagonal is the histogram of gradients for the respective element and the numbers in each box. Pearson correlation is shown in the upper-right portion of the figure while coefficient averaged over eight models for the individual correlation plot [53].

Integration of practical, relevant applications and properties ensue the High-entropy materials like a hot research area. The empirical-based frameworks rely on complex trial and error frameworks or physical intuition. A plethora of computational frameworks is dependent on computational capacity and empirical data.

Material science with the usage of ML can reduce the cost and fasten development. This study also proposed the ML method to predict synthesizability by leveraging compositional attributes and thermodynamics of a given. In the end, 70 new compositions were assessed to predict the entropy-forming capacity. Thus, a total of 108 chemical parameters have been evaluated for the target variables from the densities functional theory (DFT) data sets using an ML-based random forest model to predict EFA, as shown in the figure. Table 2 presents the identification of the essential features for predicting EFA.

The machine learning model has predicted the values of EFA against the random forest fit having chemical parameters, and eight parameters of CALPHAD were assessed for the known EFA from DFT [54]. As a result, the best-performing parameters from the machine learning model and chemical parameters are shown on the left side. Similarly, ten top-most parameters, including CALPHAD parameters for the ML data, are shown on the right side. To attain accurate predictive results, CALPHAD and chemical parameters based on ML models parameters depend on identical properties, including ionic character electronegativity electron orbitals. The value of composition-weighted average is represented with avg(x), and average deviation is represented with avg. dev(x). These values were computed employing the vector of elemental values for all the compounds. The minimum value of each compound is represented with min(x). Similarly, the maximum value is represented with max(x), and the fraction-weighted mean is denoted with fwm(x). The notation * is used to represent the parameters from CALPHAD. The correlation among the best two features and EFA decline in the ability of entropy-forming.

In higher EFA values and increasing liquidus temperature, a positive correlation exists. Blue dashes represent trendlines. Comparing EFA with liquidus temperature, ten compositions overlap entirely. For a given composition, CALPHAD is used to derive liquidus temperature, providing exciting patterns from the magnitude of anticipated EFA according to the contemplated composition. The compositions with top EFA values exist quite far from the trendlines, indicating the dire requirement of multi-variable approaches to discover useful compositions.

Now we summarize the existing machine learning-based in Table 3.

Models for modeling mechanisms are based on AI or statistics, but ML is a subset of AI; therefore, statistical methods also consider statistical methods the subset of ML. In contrast, ML algorithms are intelligent that improve automatically by experience harnessing training data to reveal trends from the data. Following is the explanation of different machine learning techniques grouped according to their type. In supervised learning, the training data was considered an input for the model training during the supervised learning process; thus, the outcome of interest is known. Many techniques focus on classification and regression models within this learning technique. The regression model results in numerical values. On the other hand, the classification model results in categorial values, i.e., yes or no. Unsupervised learning techniques are based on the outcome of interest that is not known. The renowned methods are dimensionality reduction and clustering. The clustering models involve Gaussian mixture modeling, spectral and K-means clustering, whereas principal component analysis and independent component analysis systems fall under dimensionality reduction models.

There is a research gap in the existing literature on material modeling techniques; for instance, AI and ML fields have been matured for the past decade, and a lot of contribution has been made, which is never attempted in material science. Therefore, we present future perspectives and key directions for future research for material-based modeling mechanisms.

- Deep learning is explored for modeling mechanisms; however, deep learning-based optimization mechanisms must be explored for stable material and material with maximum performance index properties.
- Existing modeling mechanisms are based on images datasets, and the regression datasets should be publicly available and explored.
- The AI-based mathematical programming should be applied throughout the material s life cycle.
- Data sampling and synthetical data should be generated using AI-based modeling mechanisms to improve the performance of material discovery and other applications.
- There is a vital need for an AI-based scientific platform based on leveraging ML and physical mechanisms.
- Need of adaptation of usability and DIY paradigms in the modeling mechanisms.

## 4. Simulation Tools and Results

Novel materials define the progression of cultures, from the ancient to the modern-day. In addition, hundreds of thousands of functional materials are significant parts of advanced technologies and infrastructures. However, it is difficult to predict an exact property and process structural relation for designing new materials with distinct properties instantly and precisely. One reason for this is the high dimensionality of features in material design, including materials’ intrinsic information and extrinsic synthesis processes’ information. The second reason is the huge material design space containing many possible materials that are difficult to select. Thirdly, the absence of specifically associated science of complex material systems. These all the analysis challenges are related to the complex management of material data, comprehension, and prediction, which surpass human capability. However, simulation tools and database construction and management tools can be developed to address these challenges. Although in literature, there are many machine learning-based simulation tools developed, this Section briefly discusses designs of well-known simulation tools and their results published in high-quality journals.

### 4.1. ElemNet

**Figure 8:**
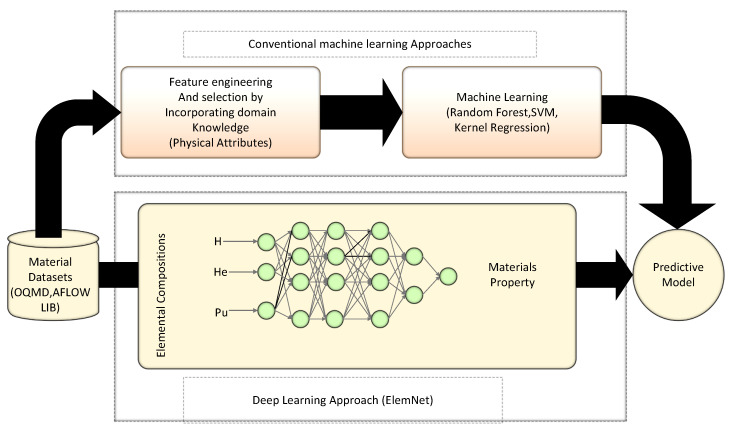
Comparison of deep learning approach with conventional ML approach for prediction of materials properties.

Deep learning the chemistry of materials from the only elemental composition. Traditional machine learning-based models to predict properties of the material over elemental compositions [55]. The study has also suggested a deep learning approach by bypassing manual feature engineering that demands domain knowledge to attain more accurate results with the help of using a minimal amount of training samples. The authors have named their proposed model ElemNet, which is based on the design and implementation of the deep neural network model. The model has the potential to automatically locate chemical and physical similarities and interactions among varying elements employing AI to predict materials properties with enhanced speed and accuracy. Figure 8 presents a comparison of the deep learning approach of the ElemNet with the conventional ML approach for the prediction of materials properties. The outcomes revealed that the ElemNet holds the potential to execute robust and fast screening for novel material candidates in high dimensional combinatorial space in which a plethora of chemical systems was predicted, which can ascertain some unidentified compounds. 

ElemNet is a model that shows a deep neural network-based framework to locate chemical and physical interaction and similar patterns autonomously. The model is permits robust and rapid screening for novel material candidates among the combinatorial space. The comparison was drawn between the deep learning model and traditional machine learning techniques to predict the properties of the materials. A plethora of chemical frameworks which may have some unidentified compounds are predicted by ElemNet. The manual or cognitive feature engineering process could be bypassed by a deep learning-based framework. These frameworks require domain knowledge and acquire good results with the mere use of training samples. Table 4 presents benchmarking of the deep learning model–ElemNet–against conventional ML approaches.

Comparison of deep learning prediction models with other machine learning-based prediction models for materials properties. The conventional machine learning-based frameworks are used to forecast the behavior of materials properties that denote the material’s composition for the model input syntax, based on the process performed via manual feature engineering techniques. The human interpretation and anticipated domain knowledge are used for the selection process by calculating the constituent elements’ physical and chemical parameters. Table 5 presents ElemNet architecture detailed configurations.

The proposed predictive deep learning-oriented framework tends to learn using better speed and accuracy than traditional machine learning models to forecast material properties, including formation enthalpy out of their elemental compositions. In the end, the comparisons were drawn between ElemNet and traditional machine learning models. The comparisons revealed that the deep learning-based ElemNet model outperformed the conventional ML models. This is because the traditional models depend on the computation of the physical attributes [55]. The deep learning model is formed using multiple layers formed using neurons, focusing on finding the potential predictive model for the formation enthalpy. The authors have performed various experiments to discover the hyperparameters space and the best DNN framework. The 0th layer is the input layer; positions and types of a varying range of dropouts and complete layers are shown. The deep learning model considers ReLU as an activation function.

### 4.2. Matminer

**Figure 9:**
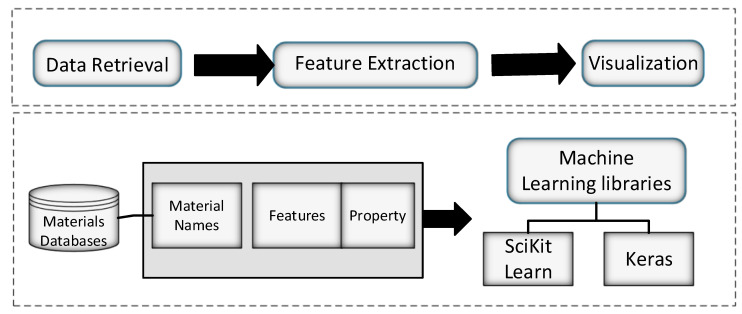
Overview of matminer simulation tool.

Since data sets pertaining to materials hold diverse nature, data mining and artificial intelligence-based methods play a vital role in material-based predictive analysis. An open-source software named matminer was designed to assist in trend analysis and prediction of material properties. Figure 9 presents the matminer tool design, which was developed using a python-based framework that provides different modules to process extensive amounts of data from explicit mediums. These mediums rely on Materials Data Facility databases, Materials Project, Materials Platform for Data Science, and Citrination. The framework also provides feasibility in providing API to execute code using a feature extraction library, designed explicitly for materials-based predictive analysis. The feature extraction frameworks utilized 47 different parameters related to featurization to derive multiple descriptors and incorporate them into math functions. In the end, the analysis outcomes are shown using visualization that offers different types of data plots. The functions are combined with machine learning and AI-based data analysis packages designed and employed by data scientists. The study has recapitulated the logic and structure of the matminer and delineated a summary of different modules [56].

The main contribution of matminer is to help users acquire extensive data from identical data sources. It forms data representations by transforming the raw data from the extracted features to develop useful visualizations that can reveal insights and integrate the useful machine learning modules in the domains of materials. Matminer has addressed various issues that arise while performing data-drive-based studies, understanding the Application Programming Interface (API) for all the data sources. Also, the pre-processed data introduces a lot of complexity while forming new machine learning frameworks. It has an interactive interface that models the expansion of the API interactions, which provides feasibility to the user in maintaining and querying comprehensive data into the standard pandas-based data format. Matminer implemented a total of 47 varying feature extraction modules. The model produces several physically relevant descriptors that can be tuned and processed by machine learning models. Moreover, the model has various pre-defined functionalities of visualization that can be used to discover relationships among attributes of the data. Matminer interacts with sci-kit-learn and python libraries of python. Moreover, it also implements the library of feature forming techniques and contains techniques that can help in information retrieval and visualization.

Table 6, lists many publicly available databases containing a large number of material structures and properties.

The existing simulation tools face almost the same problem as traditional AI mechanisms face in exploring and realizing AI in real-life applications. Therefore, new simulation tools should be developed to address data collections, data pre-processing, and modeling mechanisms. Now we present future perspectives and key directions for future research for material-based simulation tools developed.

- Systematic frameworks should be used to handle the repetitive tasks of the simulation tool.
- Traditional data science libraries should be tested and adopted in the simulation toolbox.
- General-purpose feasibility and testing mechanisms should be introduced for material performance testing in the simulation tools.
- Data sampling and synthetical data should be generated using AI-based modeling mechanisms to improve the performance of material discovery and other applications. Relationship analysis mechanisms based on AI can be explored for high-performance index properties material discovery.
- Parsing and composition assessment algorithms should be implemented in the simulation tools to explore the complex big chemical data.

## 5. Commonly Used AI-Based Materials Science Applications

The need for AI applications in the simulation and exploration of novel materials increases. Materials design based on AI analysis is foreseen to lead to innovative materials and reduce the development cost in terms of time and resources. However, the scientific community identified many limitations of advanced materials discovery and application based on AI and advanced machine learning techniques. For instance, there are several issues related to computational simulation, and the structures of the materials involved require high-performance index properties. Therefore, advanced materials research based on the convergence between AI techniques and experimental mechanisms is needed to produce the basic comprehension of the input parameters conditions and performance index properties. Picking the most-suited machine learning algorithm plays a crucial role in building a machine learning model as it dramatically impacts the model’s accuracy and generalization capability. However, no machine learning algorithm can be deemed ideal for all problems since each has its own merits in terms of applicability.

ML algorithms are categorized into four categories, as per their utilization in material sciences. The categories include regression, probability estimation, clustering, and classification. Probability estimation algorithms are primarily used to discover new materials. On the other hand, material property prediction on the macro, and micro levels is made using regression, clustering, and classification models. Moreover, machine learning systems are integrated with different optimization systems [57], including PSO, GA, or SAAs typically harnessed for optimizing the model’s parameters. In addition, these optimization models may also be utilized to perform various optimization problems like optimizing materials properties and spatial configurations. So far, we have demonstrated the role of machine learning and AI paradigms in diverse disciplines in general and specifically in material science. Let us shed light on contemporary machine learning-based state-of-the-art in material science.

Study [58] proposed developing prefabricated ceramics utilizing Machine Learning (ML). The model was trained by predetermined element analysis data combined with a self-learning algorithm to explore high-performance prefabricated ceramics in thermo-mechanical conditions. First, parametrical generation of topologically interlocked panels is performed. Then, a finite amount of developed prefabricated ceramics pointed to a thermal load is analyzed. The multilinear perceptron-based training is performed to predict the thermo-mechanical performance of prefabricated panels with the number of blocks and different interlocking angles. The formed feed-forward artificial neural network model resulted in a fillip to the prefabricated ceramic model efficiency and opened up new vistas for managing the performance for a plethora of high-temperature applications. For each of the 3 × 3, 5 × 5 and 7 × 7-block prefabricated panels, 100 random designs were examined by FEA.

The interlocking angles in the models have a varying range from 5° to 25°. Therefore, the relationship of input features to outputs is scrutinized at the pre-processing stage. The pre-processing steps, including normalizing and scaling, helped model convergence and made the training process less sensitive. The hold-out method was used for model evaluation wherein 10% of the data was picked on which 5-fold cross-validation was applied to preclude over-fitting. The study established that the prefabricated ceramic panels with the ML helped to engineer the patterns. The outcomes yielded 30% enhanced results for frictional energy dissipation and 7% in the sliding distance of the tiles, and an 80% reduction in the strain energy, which causes the high safety factor and the structural failure delay compared with the plain ceramics.

Another study [59] has proposed three different models, including multiple linear regression model (MLR), ANN, and adaptive neuro-fuzzy inference system (ANFIS), to forecast the 28 days compressive finding of concrete with 173 different mix designs. The model training and testing were done using MATLAB programming conditions. In the end, the comparisons were drawn between the three implemented models. The outcomes yielded that ANN and ANFIS validate the reliable evaluation of the compressive power of concrete with distinct mix models, but the multiple linear regression algorithm is not adequately viable in this domain due to non-linear relationships among the concrete mix parameters. On the other hand, the integration of fuzzy logic and neural network, i.e., ANFIS, can form mapping relationships among input and output variables according to human expertise.

**Figure 10:**
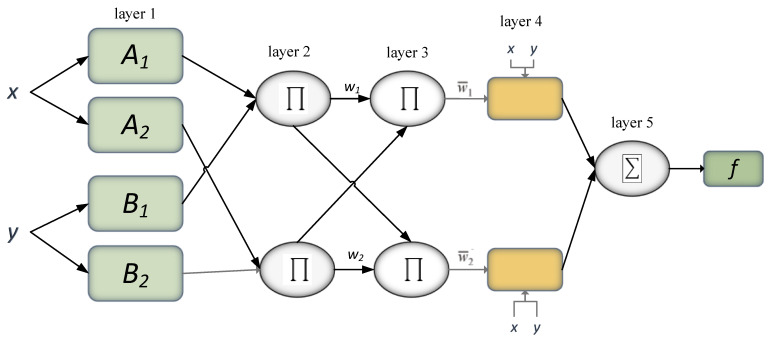
Scheme of ANFIS architecture for forecasting compressive power of concrete.

Furthermore, ANIFS holds the potential to locate interpretable IF_THEN rules that improve the model’s performance in comparison with other models [3,23,24]. The design of an ANFIS model along with two input parameters is shown in Figure 10. In the end, the sensitivity analysis (SA) for two varying sets of features on the concrete compressive power prediction is conducted. The outcomes yield that the concrete compressive power prediction performance is contingent on the number of input features. This study [60] has proposed the primary scrutiny of data set encompassing more than 10,000 observations) of calculated compressive power from actual (building-site) amalgams and their associative actual amalgam quantities. Extrapolative designs are applied to assess the nexus among the amalgam design variables and strength, thereby computing the approximate (28-day) power. These models were also used in a laboratory-based data set containing power measurements obtained. A comparison is drawn between the functioning of the designs across both data sets. Moreover, to demonstrate the significance of such methods beyond mere power projections, they are harnessed to formulate optimal concrete amalgams, reduce expense, and include CO_2_ impact while fulfilling imposed target power.

In another study [61], the authors have demonstrated how the new compound exploration procedure through ionic replacements can be designed using a mathematical model. They have proposed a probabilistic model determining the probability for ionic species to replace each other while maintaining the crystal structure. Each compound comprising (xi 1, xi 2, xi 3, xi 4) as ionic species, the probability of creating a new by replacement of a, b, c, and d for xi 1, xi 2, xi 3, and xi 4 is assessed by calculating p(a, b, c, d|xi 1, xi 2, xi 3, xi 4). If this probability is greater than the threshold value, i.e., σ, the replaced structure is contemplated. The training is performed on an empirical database of crystal structures and may be utilized to propose new compounds and their structures quantitatively. The projecting strength of the system is illustrated using cross-validation on quaternary ionic compounds. The different replacement rules entrenched in the design were assessed and compared to some of the conventional rules utilized by solid-state chemists to suggest novel compounds (e.g., ionic size).

**Figure 11:**
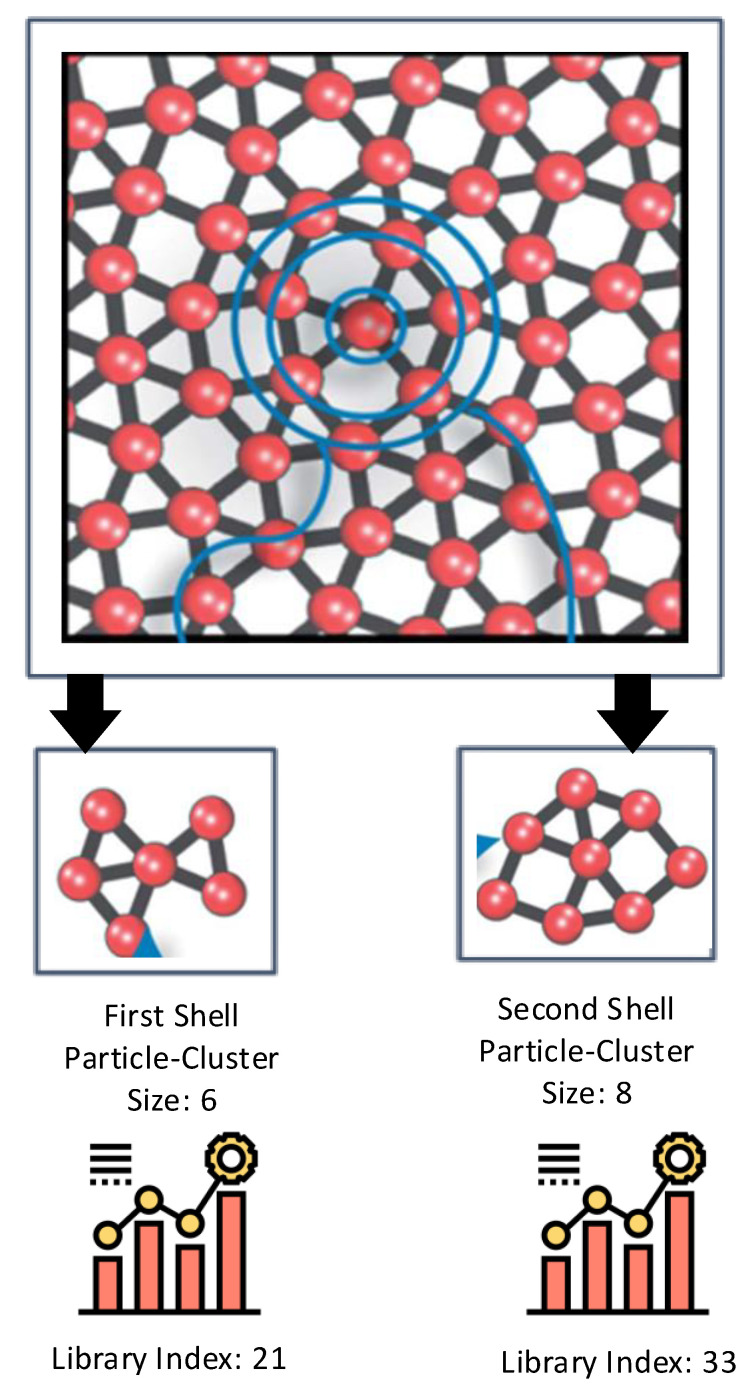
Automatic recognize new crystalline structures from big data sets of coordinates.

**Figure 12:**
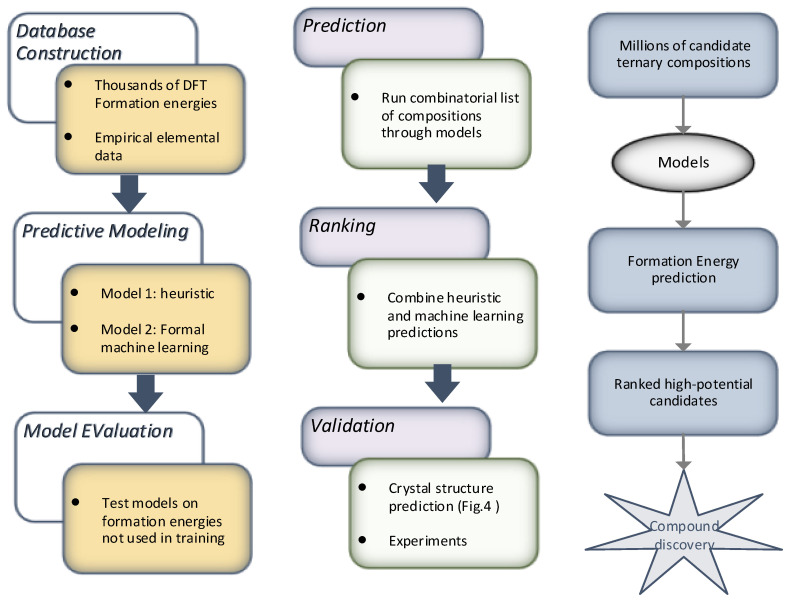
ML model from a database of thousands of DFTs calculations.

Study [62] presented a technique to automatically recognize new crystalline structures from big data sets of coordinates. The technique relies on machine learning and shape matching algorithms to extract, classify, and group local structures into common crystals. This is done by following a pattern analysis-based hierarchy. The model was evaluated on two different data sets encompassing simple and complex crystals, including quasi-crystals. The authors demonstrated how phase drawings could be automatically created and identified a crystal phase missed in prior analyses. The outcomes suggest that incorporating machine learning and shape matching algorithms for analyzing quickly formed databases hasten the identification of novel crystal materials and structures. The approach relies on two-particle clusters formed using the first and second shell cutoff radius, as shown in Figure 11. It can be inferred from the figure that there exist two methods of analyzing the type, size, and comparison to a cluster type library harnessing Fourier coefficients. The outcomes revealed that the scheme is best for soft matter systems wherein particle interactions can be intricately tuned and devised to form the self-assembly of mesoscale materials with exotic structures. Another study [63] devises a machine learning-based model for material discovery harnessing a vast volume of data encompassing thousands of density functional theory (DFT) calculations. The authors claim that the subsequent model does not require any other input, with six orders of magnitude less computer time than DFT, and has adequate potential to forecast the thermodynamic stability of arbitrary compositions. The model was harnessed to scan candidate compositions of around 1.6 million for novel ternary compounds, resulting in 4500 predictions of new stable materials. The overall flow of the model for material discovery is shown in Figure 12: part (a) denotes the formulation and evaluation of experimental and machine learning models from input quantum mechanical energetics, and part (b) shows the recognition process of new ternary compounds. The empirical and machine learning models were utilized, and a combinatorial list of ternary compositions was processed. Finally, these two models were mingled to order the compositions based on the probability of forming compounds. The results suggested that the approach can be helpful to other descriptors of interest to enhance the performance for materials discovery.

Another interesting study has used machine-learning algorithms to train the reaction data for predicting the reaction outcomes for the crystallization of templated vanadium sillenites. The parameters having information about ‘dark’ reactions as unsuccessful or failed hydrothermal syntheses were gathered from archived laboratory notebooks. Further details on basic notebook information using chem-informatics methods are incorporated by adding physicochemical property. The resulting data was then harnessed for training the machine learning-based model to forecast reaction success. Their proposed machine-learning model outclassed conventional human methods and accurately anticipated conditions for novel organically templated inorganic product formation by achieving 89% accuracy for hydrothermal synthesis experiments.

Furthermore, overturning the machine-learning model shows novel hypotheses regarding the essentials to formulate the product successfully. The authors constructed the ‘model of the model’ by re-interpreting the support vector machine models as a decision tree encompassing the IF-ELSE-based rules. The complete version of the vanadium-selenite branch of the tree envisioned in different colors specifies traditional human methods. The green lines indicate large single-crystalline products, and the blue lines represent polycrystalline products. The outcomes suggest that the model tends to accurately predict crystal formation conditions compared to the human methods, irrespective of the structural similarity of the templating amines to known examples in the database. Another study [64] has proposed an unsupervised machine learning model that finds the crucial identical patterns among the merge, allowing reported crystalline inorganic materials. The study suggests prioritizing quaternary phase fields comprising two anions for the sake of synthetic exploration to locate solid lithium electrolytes in a collaborative framework, which results in Li_3.3_SnS_3.3_Cl_0.7_ material discovery. The interstitial site combination in this defect stuffed wurtzite permits a low-barrier ion transport pathway in hexagonal close-packing.

**Figure 13:**
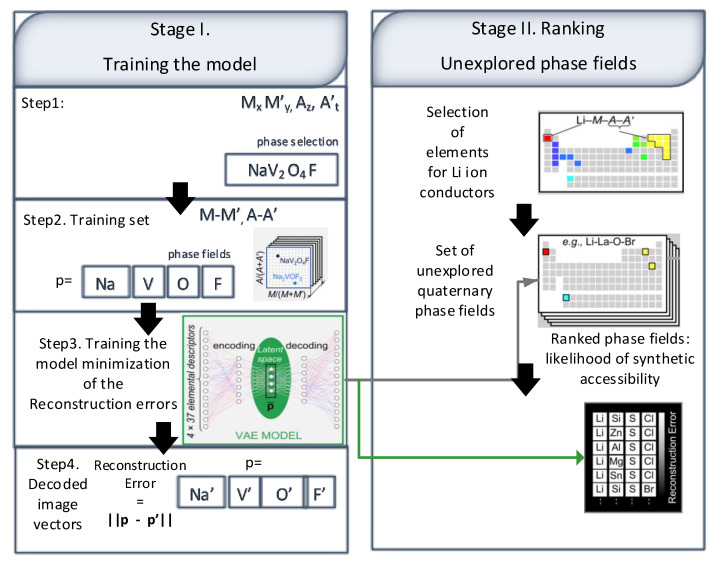
Unsupervised machine learning captures the complex patterns of similarity between element combinations.

The model was trained using phases containing 2021 MxM′yAzA to assist the prioritization of the candidate phase fields, as shown in step 1 of Figure 13, and step 2 is performed to show the concentrations. The variational autoencoder (VAE) mechanism was adopted to reduce the dimensionality from an unsupervised neural network, as shown in step 3 Figure 13**.** In the third step, a similar non-linear pattern is detected from the highly dimensional unsupervised instances of the data. The reconstruction error was computed using Euclidean distances, which was later minimized using VAE based training encoding method, as shown in step 4 of Figure 13. The study [65] has suggested a deep learning-based forecasting model to identify mechanical properties of industrial steel plates such as elongation (EL), yield strength (YS), impact energy (Akv), according to the process parameters along with raw steel combination. The model was later applied on a real steel manufacturing plant online. The proposed optimal deep neural network (DNN) model comprises 27 input features, having 2 hidden layers spanning 200 nodes and four target variables.

**Figure 14:**
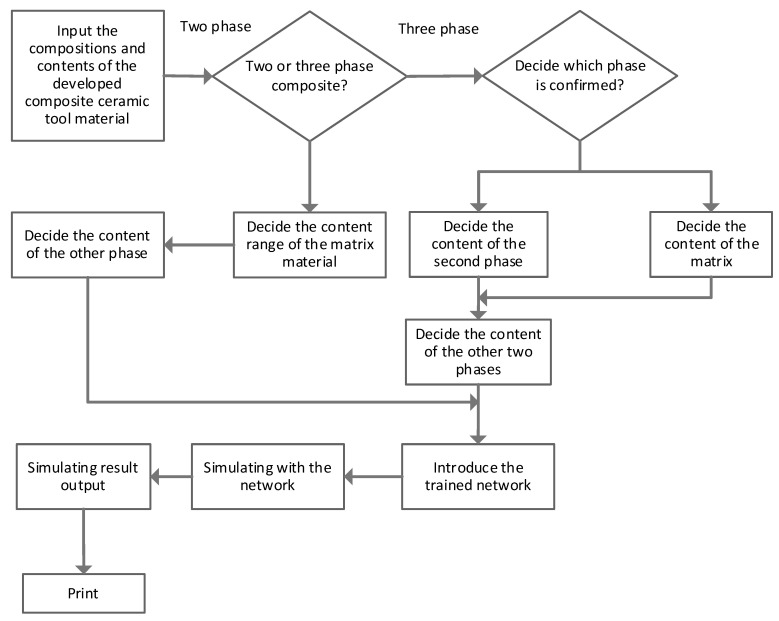
The flow chart for predicting the mechanical property. The model has investigated the non-linear relationship among the raw material’s content composition, component, and the fracture complexities of the composite ceramic and flexural strength. The proposed ANN model predicted the mechanical characteristics of the alumina matrix ceramic tool. The neural network model was trained using a toolbox available in MATLAB (MATrix LABoratoryMA software). The two and three-phase composite ceramic tools of the mechanical properties like Al_2_O_3_–(W, Ti)C and Al_2_O_3_–TiC–ZrO_2_ were predicted to verify their proposed model.

The model used an Adam optimizer, and the starting value of the learning rate was fixed as 0.0001. The model employed the Z pre-processing method to make an optimal model with R2 as 0.907. The DNN model was evaluated using RMSE MPA percentage error which resulted in 21.06, 16.67, respectively. The RMSE percentage error resulted in 4.7% for YS, 2.9% for ultrasounds testing (UTS), 7.7% for EL, and 16.2% for Akv. The outcome results revealed that the model outperformed the existing machine learning models. The insights of their proposed model were further revealed using different local linear models by establishing a connection among mechanical properties and process parameters. The designed model was later applied to a real scenario where online supervision and steel mechanical properties were controlled. The deployed model was harnessed to monitor the creation of desired steel plates and mechanical properties. The study [66] has introduced an ANN model and applied it for ceramic material detection. The overall flow of the model can be seen in Figure 14, which shows matrix material’s content range can be acquired if there are two phases in ceramic tool material.

On the other hand, the second phase can be fixed if there are three phases in composite ceramic tool material. Then, the remaining two steps can be amended and optimized to forecast the mechanical characteristics for varying content conditions. The study’s outcomes revealed that the ANN-based resulted model is quite helpful in simulating the composition content and predicting the mechanical aspects of the ceramic tool. Another study [67], backpropagation artificial neural networks (BP-ANNs), and orthogonal experiment design (OED) model have suggested addressing multi-purpose objective problems raised because of the preparation of alumina slurry. The relationship between the slurry model’s influencing factors and extrusion parameters harnessing the integrated model. The model’s effectiveness was ensured by consistently the foretold optimal values with the empirical results. The outcomes suggest that the Alumina slurry model is helpful and holds significant shape retention and extrusion properties for 3D printing. The model was presented to be used to other various other multi-objective problems about ceramic materials. A linear strain distribution is considered toward the thickness of the passive plate.

In the paper [68], a linear strain distribution is assumed across the thickness of the passive plate of the lead zirconated titanate (PZT) actuator given the mechanical properties such as Young’s modulus, and Poisson ratio of the actuator and the passive plate are close. An analytical equation for the passive plate deflection is derived from this assumption. The analytical result shows excellent agreement with experimental data and the results from limited element simulation. Based on this analytical model, the effects of several vital parameters and non-dimensional variable groups on the actuator performance have been inspected. These parameters and variables include the dimensions and mechanical properties of the PZT disk, the passive plate, and the bonding layer material.

The critical factors in establishing the strengthening energies of solutes in varying metallic GBs were predicted using three machine learning models, including SVM with radial basis function (RBF) kernel, SVM, and artificial neural network (ANN). The historical density functional theory calculations containing 142 strengthening energies were employed to train ML models. Among all the models, the non-linear kernel-based SVR model outperformed the other two models regarding atomic size and bond-breaking effect by attaining r2 as 0.889. The prediction output was significantly enhanced by only employing bond-breaking impact. Furthermore, mean impact values’ scrutiny was carried out to reveal quantitatively explore the relative significance of all the input parameters to attain the valuable prediction output.

ML-based models, including SBL, clustering analysis, and classification for alleviating the supply chain risk, and finding variations and validation and consistent material quality is presented by [69]. Moreover, a certificate of assurance and eCoA SPC control systems were introduced to validate the quality of raw materials. The main focus of the study is to attain accurate in-time monitoring of the capability of the supplier process by reducing foundry manufacturing risk via executing the optimal quality control mechanism to monitor control raw material COA and timely identification of raw materials encountered during the inspection stage at the foundry site.

The study [70] focuses on determining the ultimate tensile strength pertaining to the strain hardening of a material. In other words, the authors developed a methodology by dividing a data set into different categories randomly. Then, a fully connected topology was formed wherein further training and prediction rounds were carried out, and average performance was acquired. By doing this, the behaving pattern of the network was revealed that the network has 150 perceptron’s in the hidden layers resulting in less than 4% predictive error. Another study [71] has proposed an ANN-based approach to model the micromechanical behavior of CMCs. The ANN model was learned on the complex multi-parametric interaction between multiple microstructural features by considering the example of SiC/SiC ceramic composite. First, the macro-mechanical behavior of the SIC (matrix)/SiC (fiber) composite was determined with the help of ANN. Then, a micromechanical finite element analysis is carried out using the model’s training samples, resulting in realistically interfacial debonding and sliding. Finally, the network has learned and evaluated by predicting the behavior of the composite for novel specimens. This study has critically addressed the steps involved in ANN training, such as data set preparation, configuring network, training, and testing.

Similarly, machine learning and artificial intelligence-based tools have been utilized to form mechanical material models and temperature-dependent thermal for structural steel [72]. As a result, steel structures’ structural and thermal response was predicted. The outcomes were evaluated with the help of various case studies conducting harnessing a highly non-linear finite element model designed in an ANSYS simulation environment.

This study has also employed compositional and thermodynamic features of a given material to predict the synthesizability, such as the entropy-forming ability of the disordered metal carbides using machine learning-based methods. First, the relative significance of the compositional and thermodynamic attributes was scrutinized for the prediction. Then, the density functional theory was then adopted for ML predictions wherein the model’s suitability was delineated. In the end, the model was employed for predicting the entropy-forming capability for 70 novel compositions. The experimental synthesis and different density functional theory computations were utilized for evaluating the prediction accuracy. Specifically, seven compositions were picked as they hold all three Group VI elements (Cr, Mo, and W), which do not provide room temperature-stable rock-salt monocarbides—adding the Group VI elements into the rock-salt structure to stipulate the situation to tune potentially material output and electronic structure. Another study [73] has presented a machine learning-based data-fusion model that served for nondestructive testing applications in the context of characterization and detecting the flaws. The features were derived from UTs and eddy current testing (ECT) signals. The Partial Least Squares were utilized for feature extraction. The proposed data-fusion model was evaluated to know the performance for characterization and localization rather than one inspection technique only as done by other similar studies.

**Figure 15:**
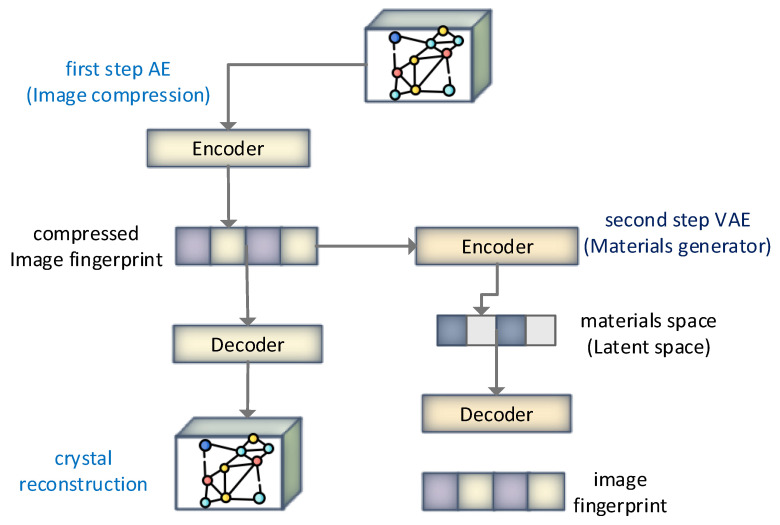
Hierarchical two-step image-based materials generator. To summarize the applications of ML and AI in material science, it is worth mentioning that scholarly and industrial researchers have applied machine learning techniques for data analysis. Using algorithms that learn based on data, machine learning methods automate building analytical models. Classification and regression are areas in which machine learning has good applicability, and therefore it has played a critical role in many fields of Material science [75,76,77]. Materials science has been using machine learning applications since the 1990s. For instance, the machine learning method predicted the corrosion behavior and tensile strength in ceramic matrix composites [29,30]. Therefore, machine learning has been used to study a wide range of topics in materials science, such as the discovery of new materials and the prediction of material properties. Table 7 summarizes machine learning applications in material science categorized into the material field, application, description, and the AI and ML mechanism used.

A latent space representation-based model was presented in [74], wherein the continuous representation of materials was learned, and the model was built for new material discovery. The capability of autoencoders to form empirical materials is delineated using vanadium oxides through the reidentification of empirically aware structures during the model training without their consideration. The overall flow of the model is shown in Figure 15. Around 20 thousand hypothetical materials were formed, resulting in various novel metastable V_x_O_y_ materials which could be synthesizable. The comparison was drawn using GAs, which resulted in the computational ability of the generative models holding the potential to scrutinize the chemical compositional space efficiently via learning aware materials’ distribution to predict crystal structure. The authors claim that the proposed model is quite useful for inorganic functional materials using generative models from the machine learning paradigm.

**Table 7:** Summary of machine learning applications in material science.

Material Field	Application	Description	AI and ML Mechanisms
Ceramics	Design of architectured ceramics	Accelerated design of architectured ceramics with tunable thermal resistance via a hybrid machine learning and finite element approach	ANN
Composite	Compressive power of concrete [59]	Forecast the 28 days compressive finding of concrete with 173 different mix designs	ANN, ANFIS
Solid-state	Crystalline structures recognition [62]	Recognition of new crystalline structures from big data sets of coordinates	Probabilistic model
Thermodynamic	Thermodynamic’ stability forecasting [63]	Thermodynamic’ stability for-casting model from a database of thousands of DFTs	Predictive modeling
Inorganic materials	Patterns of similarity between element combinations [64]	Unsupervised ML model for identifying patterns among the element combinations of crystalline inorganic materials	Unsupervised ML model
Ceramics	Ceramic material detection [66]	Investigating the non-linear relationship among raw materials content composition, component, and the fracture complexities of the composite ceramics	ANN
Ceramics	Mechanical property prediction [67]	Simulating the composition content and predicting the mechanical aspects of the ceramic tools	ANN
Solid-state materials	Two-step image-based inverse design of functional materials [74]	Inverse design and latent space-based representation-based ML model for functional materials	ML-based inverse design
Clay	Clay prediction [78]	ML model deployed on a low-cost portable device for clay prediction	Multi-variate calibration techniques
Soil matrix	Soil organic carbon mapping (SOC mapping) [79]	SOC mapping based on remote sensing data based predictive modeling	SOC prediction models
Painting materials	Colorimetric analysis materials [80]	Photometric UVC based on PLS regression for colorimetric analysis materials	PLS regression

## 6. Conclusions and Future Research Direction

The use of AI methods in materials science has received notable attention from the scientific community. Many ML-based methods have been presented to analyze big data retrieved from material databases and datasets to extract hidden knowledge and its utilization in the relevant paradigms. These tools provide correlations between many complexes and interrelated structures of materials composition. However, there is a lack of a detailed analysis of the existing material discovery methods to have precise insights related to contemporary state-of-the-art benchmark data set, pre-processing, prediction algorithms, and simulation methods. This study presents an in-depth analysis of the datasets available for material science applications, data pre-processing and AI-based modeling techniques, and materials simulation tools. The study’s outcomes revealed that deep learning-based methods had shown significant improvements and potential for predicting compositions, processes conditions, and performance properties of materials to identify the changes in specific parameters for modeling materials behavior. Moreover, advanced AI mechanism-based models have been discussed in detail for designing and optimizing properties prediction for advanced materials discovery.

However there is a research gap of AI mechanisms exploration for the whole life cycle of material science. With the expansion of computing, interdisciplinary research to the material science subfields, and different stages of material discovery and assessment, promising future directions have been discovered. The research gap should be bridged using AI and advanced computing techniques through convergence mechanisms. New research methods should be introduced to effectively combine the AI and ML mechanisms in the life cycle of material discovery to accelerate the entire process. Systematic frameworks should be proposed based on AI, ML, and advanced data science mechanisms to supersede different computationally expensive modeling mechanisms and simulations tools. Moreover, Composition assessment and material discovery mechanisms can be proposed based on the cutting-edge AI mechanisms.

## Figures and Tables

**Table 1:** Summary of datasets based on names, material properties, dataset size, and AI tasks.

S. No	Dataset Name	Material Property	Dataset Size	AI Task Type
1	Pilania_double_perovskites_clean	Bandgap (Eg)	1306	Regression
2	Pilania_Polymers_data	Atomization Energy Bandgap Electron Affinity Formation Energy Lattice Parameter Electronic Dielec Const	175	Regression
3	Pilania_Polymers_data_Spring_Const clean	Spring Constant	174	Regression
4	Pilania_Polymers_data_total_Diele Const_clean	Total Dielec Const	174	Regression
5	Pilania_superlattices	Interfacial Energy Lattice Parameter Formation Energy	1250	Regression
6	Pilania_superlattices_GGA_Band_ Gap_clean	GGA Bandgap	1249	Regression
7	Pilania_superlattices_HSE_Band Gap_clean	HSE Bandgap	121	Regression
8	Pilania_superlattices_elastic_cons	Elastic Constants: c11, c12, c13, c33, c44	987	Regression
9	Wei_composite_materials	Effective Thermal Conductivity	720	Regression
10	Wei_porous_media	Effective Thermal Conductivity	374	Regression
11	Zeng_elastic_prop	Elastic Moduli: Shear Modulus (G) Bulk Modulus (K)	5518	Regression
12	Bala_classification_dataset	Curie Temperature (Tc)	192	Classification
13	Bala_regression_dataset	Curie Temperature (Tc)	132	Regression
14	Lee_band_gaps	Bandgap (G, Wo)	270	Regression
15	(Li_DFT_and_features_clean and Li_DFT_dataset_clean	Ehull	1925	Classification
16	Mannodi_polymer_diele	Electric Dielec. Const. Bandgap Lonic Dielec. Const. Total Dielec. Const.	284	Regression
17	Seko_melt_temps	Melting Temperature (Tm)	248	Regression
18	Wu_DFT_Eg_dielec_consts	Bandgap Electric Dielec. Const.	155	Regression
19	Wu_Exp_Tg	Glass Transition Temp (Tg)	262	Regression
20	Zhuo_classification_data	Bandgap (Eg)	6354	Classification
21	Carrete_therm_conduct_train_clean	Lattice Thermal Conductivity (kw)	30	Classification
22	Liu_Tg_AsSe_glass	lass Transition Temp (Tg	12	Regression
23	Rajan_Mxene_data	Bandgap (Eg)	70	Regression
24	Wu_Exp_die lec_const	Dielectric Constant	58	Regression
25	Wu_loss_tang_l OOHz	Dielectric Loss Tangent	48	Classification
26	Wu_loss_tang_kHz	Dielectric Loss Tangent	44	Classification
27	Xue_thermal_	Therma l Hystersis	22	Regression

**Table 2:** Identification of the essential features for predicting EFA.

Predictor Rank	Stoichiometric Attributes	CALPHAD
1	avg(ionic character)	avg(ionic character)
2	min(electrons)	Liquidus temperature
3	avg. dev(s-valence electrons)	range(electronegativity)
4	max(atomic weight)	avg. dev(d-valence electrons)
5	max(covalent radius)	max(atomic weight)
6	fwm(covalent radius)	fwm(f-valence electrons)
7	range(Mendeleev number)	max(covalent radius)
8	avg. dev(melting temp)	max(unfilled valence electrons)
9	fwm(unfilled s-valence)	fwm(covalent radius)

**Table 3:** Predictive mechanism used in material science applications.

Methods	Category
Least-squares regression	Regression
Kernel ridge regression	Regression
Kriging or Gaussian process regression	Regression
Artificial Neural Network	Regression Classification
Support Vector Machine	Regression Classification
Decision tree	Classification
Random forest	Classification
k-nearest neighbors	Classification
Naive Bayes	Classification

**Table 4:** Benchmarking of the deep learning model–ElemNet–against conventional ML approaches.

Model	Input Type	MAE (eV/atom)	Training Time (h)	Prediction Time (s)
RandomForest	Physical Attributes	0.071 ± 0.0006	1.5	14.80
RandomForest	Elemental Compositions	0.157 ± 0.0012	1.5	2.87
ElemNet	Elemental Compositions	0.050 ± 0.0007	7 (GPU)	9.28 (CPU) & 0.08 (GPU)

**Table 5:** ElemNet architecture.

Layer Types	No. of Units	Activation	Layer Positions
Fully-connected Layer	1024	ReLU	First to 4th
Drop-out (0.8)	1024		After 4th
Fully-connected Layer	512	ReLU	5th to 7th
Drop-out (0.9)	512		After 7th
Fully-connected Layer	256	ReLU	8th to 10th
Drop-out (0.7)	256		After 10th
Fully-connected Layer	128	ReLU	11th to 13th
Drop-out (0.8)	128		After 13th
Fully-connected Layer	64	ReLU	14th to 15th
Fully-connected Layer	32	ReLU	16th
Fully-connected-Layer	1	Linear	17th

**Table 6:** Summary of publicly accessible databases and simulation tools.

Name	Description
AFLOW	Online applications for property predictions using machine learning
CALPHAD	Computer coupling of phase diagrams and thermochemistry
Matminer	Data source, descriptive and predictive analysis
ElemNet	Deep learning-based mechanism
ChemSpider	Search engine for chemistry’s structure database
Citrination	AI-Powered materials data platform
Computational Materials Repository	Repository for infrastructure framework for CMR
Harvard Clean Energy Project	Properties computation of materials
ICSD	Multiple databases targeting materials properties
MatNavi	A database of structures and properties
MatWeb	Searchable database of material properties i
NIST	Chemistry webbook
NIST Materials Data Repository	Repository for published materials data

## Data Availability

Not applicable.
